# Perinatal suicidal ideation and behaviour: psychiatry and adversity

**DOI:** 10.1007/s00737-016-0706-5

**Published:** 2016-12-28

**Authors:** Michael Nnachebe Onah, Sally Field, Jason Bantjes, Simone Honikman

**Affiliations:** 1 Perinatal Mental Health Project, Alan J. Flisher Centre for Public Mental Health, Department of Psychiatry and Mental Health, University of Cape Town, 46 Sawkins Road, Building B, Rondebosch, Cape Town, 7700 South Africa; 2 Department of Psychology, University of Stellenbosch, Stellenbosch, South Africa

**Keywords:** Suicidal ideation, Suicide risk, Pregnant women, Poverty, Correlates

## Abstract

Pregnant women are at increased risk for suicidal ideation and behaviours (SIB) compared to the general population. To date, studies have focused on the psychiatric correlates of SIB with lesser attention given to the associated contextual risk factors, particularly in low- and middle-income countries. We investigated the prevalence and associated psychiatric and socio-economic contextual factors for SIB among pregnant women living in low resource communities in South Africa. Three hundred seventy-six pregnant women were evaluated using a range of tools to collect data on socio-economic and demographic factors, social support, life events, interpersonal violence and mental health diagnoses. We examined the significant risk factors for SIB using univariate, bivariate and logistic regression analyses (*p* ≤ 0.05). The 1-month prevalence of SIB was 18%. SIB was associated with psychiatric illness, notably major depressive episode (MDE) and any anxiety disorder. However, 67% of pregnant women with SIB had no MDE diagnosis, and 65% had no anxiety disorder, while 54% had neither MDE nor anxiety disorder diagnoses. Factors associated with SIB included lower socio-economic status, food insecurity, interpersonal violence, multiparousity, and lifetime suicide attempt. These findings focus attention on the importance of socio-economic and contextual factors in the aetiology of SIB and lend support to the idea that suicide risk should be assessed independently of depression and anxiety among pregnant women.

## Introduction

Suicide has been identified as one of the major contributors to the global mortality burden (Goldsmith et al. 2002; Nock et al. 2008; Turecki and Brent 2016), and there is a growing concern over the increase in suicidal ideation and behaviour (SIB) among pregnant women (Frautschi et al. 1994; Gagliardi et al. 2014). An estimated 75.5% of suicides occur in low- and middle-income countries (LMICs) (WHO 2014). It is thus important to identify risk factors pertinent to these settings. Comorbid mental health disorders, particularly diagnoses of depression, anxiety, alcohol misuse and substance abuse have been shown to be the strongest risk factors for SIB (Dumais et al. 2005; Nock et al. 2010) in the general population, among pregnant women (Bayatpour et al. 1992; Bonari et al. 2004) and in the postpartum period (Gold et al. 2012), in both high and LMIC settings. Borderline personality disorder (BPD) is one of two DSM-IV diagnoses for which suicidal behaviour is a criterion (American Psychiatric Association 2013). Among these individuals, the behaviour manifests as severe emotional distress and inability to control behaviour (Linehan et al. 2006). Recently, however, attention is being focused on the importance of socio-economic, cultural and contextual determinants of SIB and the importance of considering suicidal phenomena independent of psychiatric disorders (Vijayakumar et al. 2005; Fliege et al. 2009; O’Connor et al. 2011; Chan 2013), even though some of the determinants may be common (Fisher et al. 2012). The focus on contextual determinants of mental health problems and SIB is important, particularly in low resource environments, where the scarcity of mental health professionals necessitates the use of suicide prevention interventions that do not only focus on the psychiatric determinants of SIB.

Globally, a range of prevalence rates has been reported for perinatal SIB. A 2004 review of 17 studies in high- and low-income countries found the prevalence of suicidal ideation among pregnant and postpartum women to range between 5 and 18% with low-income countries presenting higher prevalence rates (Lindahl et al. 2005). These findings are corroborated by more recent studies from other LMICs, with a range prevalence rates from 6.3 to 14% (Gausia et al. 2009; Huang et al. 2012; Zhong et al. 2015; Castro e Couto et al. 2016).

A South African study, using the Structured Clinical Interview for DSM Disorders diagnostic tool, found a 27.5% prevalence of any suicidal ideation among pregnant, rural women. The authors found positive associations between economic and sociodemographic factors, suicidal ideation with depression (past and present) and HIV status (Rochat et al. 2013). A further South African study found positive associations between predictor factors (younger age, unemployment, not married and positive HIV status), food insecurity, depression, hazardous drinking and suicidal ideation among postnatal women in a peri-urban setting. This study used the MINI diagnostic tool and found a prevalence of 7.6%, of which 2.8% were classified as high risk (Dewing et al. 2013).

Research from high-income countries suggests that SIB is extremely rare in the absence of major mental illness (Reinherz et al. 2006). Studies have suggested that over 90% of adult suicide deaths in high-income countries are associated with psychiatric disorders (Lesage et al. 1994; Conwell et al. 1996), notably with depressive disorders (Wulsin et al. 1999), psychotic illness (Hawton et al. 2005) and PTSD (Krysinska and Lester 2010). Similarly, the association between non-fatal suicidal behaviour and mental illness has been documented in systematic reviews (Fliege et al. 2009). Suicidal ideation has also been associated with symptoms of psychopathology, in particular depressive disorders and borderline personality disorder (American Psychiatric Association 2013).

Less attention has been paid to documenting the contextual factors associated with SIB, although this is becoming an area of growing interest. One systematic review including low- and high-income countries found that low socio-economic status and low levels of educational attainment were as significant as psychiatric risk factors in the aetiology of completed suicides (Li et al. 2011). Long-term unemployment (Milner et al. 2013) and poverty have been linked to SIB in low- and middle-income countries (Iemmi et al. 2006). A study of factors associated with suicidal ideation in the general population found that younger age, being married, negative life events and lack of social support increased the odds of suicidal ideation, although there were significant gender variations (CASEY et al. 2006). It would seem that that there are geographic variations with respect to the associations found between socio-economic variables and suicide (Rehkopf and Buka 2005) as well as ethnic variations (McKenzie et al. 2003), which highlights the need for context-specific studies in this field.

A Confidential Enquiry into Maternal Deaths in the United Kingdom found that suicide and other psychiatric disorders were the leading cause of maternal death and called into question the widely accepted belief of the protective effect of maternity (Oates 2003). A systematic review of studies in low- and middle-income countries found a pooled prevalence rate between 0.65 and 3.55% for maternal deaths attributed to suicide (Fuhr et al. 2014). Risk factors identified included poverty, lack of support, lack of trust in health systems and comorbid mental illnesses. According to models proposed by Fisher (2016), theoretically, suicide is postulated to be the endpoint of a set of multifaceted pathways which components include, among other factors, poverty, social inequality and living in a dangerous neighbourhood.

To the best of our knowledge, there has been no research on prevalence, associated risk and comorbidities of SIB among pregnant women in low-income, urban South African settings using the MINI diagnostic interview. Hence, this study aims to investigate these factors among pregnant women in Hanover Park, Cape Town, South Africa.

## Methods

### Study design and setting

This cross-sectional study was conducted in Hanover Park, among pregnant women attending the Hanover Park Midwife Obstetric Unit (MOU) for antenatal, delivery and postnatal care. Hanover Park is a low-income, residential and industrial suburb within the City of Cape Town. This area is characterised as one of the most violent communities in Cape Town with high levels of gangsterism, gun fights, rape, and alcohol abuse and drug use (DoH 2013). There are also high rates of unemployment and low rates of adult literacy (Moultrie 2004).

### Sample and procedures

We implemented a systematic sampling technique where we recruited every third woman visiting the MOU for her first antenatal care visit, regardless of gestation. We used this sampling frame (*k* = 3) after considering the average number of women that attended the MOU for antenatal care and the duration of their recruitment, interview and booking procedure at the clinic. This information was then used to estimate the average number of women recruited on a daily basis and for calculating the sample number. Women were only approached on their initial booking visit at the facility. Women who were returning for follow-up visits were not approached to participate. There was no financial incentive provided for participating in the study. We approach 559 eligible women to participate in the study. Of these, 135 (24%) declined to participate; therefore, the study had a 76% rate of agreement. A further 48 women did not complete the questionnaires in full and were excluded from the database. In total, 376 pregnant women were recruited to participate in the study.

### Measures

In order to control or limit the effect of information bias, we used data collection tools that have been validated in different settings. We also performed reliability and internal consistency tests among scale tools used. Variables that were susceptible to wide variations due to recall bias were eliminated from the study. These included household income and retrospective HIV information.

A detailed questionnaire was administered which explored participant information pertaining to demographic, socio-economic, obstetric, pregnancy and past mental health history data. Income data collected was converted to US dollars (XE Currency Data 2014). The presence of risk factors for psychological distress during pregnancy was assessed using the Risk Factor Assessment (RFA) tool which was developed by the Perinatal Mental Health Project (Honikman et al. 2012; Vythilingum et al. 2013). Perceived support from family, friends and partners was evaluated using the Multidimensional Scale of Perceived Social Support (MSPSS) (Zimet et al. 1988). This tool exhibits psychometric accuracy and has been used in South Africa among diverse populations (Williams et al. 2008; Myer et al. 2008b). Presence of intimate partner violence (IPV) among study participants was appraised using the revised Conflict Tactic Scales (CTS2) tool. This tool is a concise version of the Conflict Tactic Scales (CTS) (Reichenheim 2004) and has been used in South African settings (Gass et al. 2010; Devries et al. 2013). Food insecurity was examined using the revised six-item Household Food Security Survey Module (HFSSM) (Blumberg et al. 1999) which was developed to assess household hunger and food security in surveys. This tool collected information on the period 6 months prior to the survey. A score of 2–4 was considered as food insecure and 5–6 was considered as a more severe form of food insecurity score, labelled as food insufficiency, on the six-item scale.

The Expanded Mini-International Neuropsychiatric Interview (MINI Plus) Version 5.0.0 is a diagnostic tool and has been used and validated in several studies and in South Africa (Lecrubier et al. 1998; Myer et al. 2008a; Spies et al. 2009; Chen et al. 2010; Dewing et al. 2013). This tool is Diagnostic and Statistical Manual of Mental Disorders, 4th Edition (DSM-IV) categorised, and was used to diagnose major depressive episode (MDE), any anxiety disorder (including PTSD), alcohol abuse and drug (AOD) use, and antisocial personality disorder (ASPD) among participants. Suicidal ideation and behaviour was gauged using the MINI Plus suicide module (module C) which comprises nine questions that assess the presence of SIB, lifetime suicide attempt, frequency of suicidal thoughts and intention of suicidal act. We developed three distinct suicide nomenclatures: suicidal ideation (questions c2–c4), suicidal behaviour (questions c5–c8) and SIB (questions c2–c8), while lifetime suicide attempt was populated from question c9. Suicidal ideation included only those that had suicidal thoughts. Suicidal behaviours are those that had suicidal thoughts and had proceeded to plan or prepare or attempt suicide. SIB included all those with ideation only as well as those that planned, prepared or attempted suicide. Some of the currently suicidal women may have previously attempted suicide; however, those that had only attempted suicide in the past were removed from the current suicidality analysis. According to the wording of the MINI Plus suicide module, data was assessed for those with suicidal ideation and behaviour during the month prior to the assessment. The module has been shown to be a valid predictor of future suicidal behaviour in patients with and without a previous suicide attempt (Roaldset et al. 2012). Moreover, all single items of the scale significantly predict future suicidal behaviour (Roaldset et al. 2012). Current SIB is defined as those who, in the month prior to assessment, had thought, planned and or acted on suicidal thoughts.

All the tools used were translated and back translated from English into the other predominant local languages spoken in the area; Afrikaans and isiXhosa.

### Data collection

A registered mental health counsellor and research assistant were trained and supervised by a clinical psychologist, with research experience, to collect data. The research assistant administered the screening questionnaires and psychosocial risk instruments. The registered counsellor administered the MINI diagnostic tool. She had a 4-year tertiary qualification in psychology with 3 years of clinical experience and was able to conduct the interviews in the language of choice for the participants, using an interpreter for isiXhosa. Women who were diagnosed with psychotic disorders or exhibited suicidal ideation according to the MINI were immediately referred for further assessment and management to emergency or mental health professionals at the Community Health Centre, on the same premises as the MOU. Women with MINI-diagnosed non-psychotic disorders were offered counselling, free of charge, with the counsellor conducting the assessment. All tools were administered as an interview. Data were collected between November 2011 and August 2012.

### Data analysis

Data analysis was performed using Stata v13.1. Households were grouped based on their socio-economic status as ascertained by an asset index. An asset index was constructed due to the weaknesses observed in the household income data. These weaknesses include difficulty in collection due to recall bias and lack of sensitivity to non-cash, in-kind transfers and income in poor settings (Deaton 1997; Montgomery et al. 2000; Filmer and Pritchett 2001; Vyas and Kumaranayake 2006). Asset indexes has been used in studies in LMICs to stratify household based on their socio-economic status (Booysen et al. 2008; Harttgen et al. 2013) especially in low-income urban and rural settings (Michelson et al. 2013) and in South Africa (Kabudula et al. 2016). To construct the asset index, information on ownership of electronic equipment (e.g. microwave, washing machine, television and fridge), transport (cars), sources of energy (electricity) and bank accounts (including credit card) were pooled together. In the principal component analysis, the first component factor was used to represent the asset index. The first component factor in statistically term is defined as the weighted sum of the different assets used to measures household wealth, in order for that component to explain as much as possible of the variance observed in asset ownership between households. Based on this analysis, the study population was classified into four quartiles (i.e. least poor, poor, very poor and poorest).

Data were described using sample statistics. Internal consistency and scale reliability within assessment tools were assessed using the Cronbach’s α Statistics (Cronbach 1951). Multicollinearity was assessed among independent variables within the regression model (Chen et al. 2003). Significant associations were examined using non-parametric tests: the Wilcoxon sum of rank test, the Fisher exact test and the two sample *t* test. Bivariate analyses were performed to examine associations between independent and predictor variables. Crude logistic models were used to test association between individual predictor variables and the outcome variables of interest. This was then built into an adjusted model to assess multiple associations and control for covariates and potential confounding variables. The threshold for significance level was placed at 0.05.

## Results

Three hundred and seventy-six (376) women were sampled in the study (Table 1). Over half of the women were in the second trimester of their pregnancy. While 94% of these pregnant women were currently in an intimate relationship, 39% were married and 51% were in a stable relationship, but not married. Sixty percent of the sample was cohabiting with their partner. While 60% had education levels higher than grade 10, 58% were not working at the time of the study. While 29% of women in the sample belonged to the highest socio-economic quartile based on their asset index, only 5% of women earned a monthly salary above 500 USD. Forty-two percent of the entire sample was food insecure, while 13% indicated food insufficiency.

**Table 1:** Description of study participants

	Variable	*N* (376)	Percentage
Demographic factors	Age of woman		
	18–24 years	146	39
	25–29 years	114	30
	Above 29 years	116	31
	Gestation in weeks		
	First trimester	96	32
	Second trimester	175	58
	Third trimester	29	10
	Type of relationship		
	Married	144	39
	Stable unmarried relationship	192	51
	Casual unmarried relationship	16	4
	No relationship	22	6
	Cohabiting with partner		
	No	97	28
	Yes	209	60
	Sometimes	44	12
	Education level		
	Grade 10 or below	151	40
	Higher than grade 10	225	60
	Employment status		
Socio-economic factors	Working currently	159	42
	Not working currently	217	58
	Asset index^a^		
	Least poor	107	29
	Poor	91	24
	Very poor	92	24
	Poorest	85	23
	Individual income (monthly)		
	R0 ($0)	97	26
	R1–R1000 ($1–100)	99	26
	R1001–R2000 ($101–200)	64	17
	R2001–R5000 ($201–500)	97	26
	>R5000 ($500)	19	5
	Food security		
	Food insecure	158	42
	Food insufficient (extreme food insecurity)	50	13
Pregnancy info	Pregnancy intention		
	Intended pregnancy	137	37
	Unintended pregnancy	237	53
	Pregnancy outcome		
	Not pleased with pregnancy	295	78
	Pleased with pregnancy	81	22

^a^First component accounted for 45% of the total variation in the PCA

The prevalence of current SIB was 18% using the MINI diagnostic tool. When SIB was disaggregated, we recorded a 12% prevalence of current suicidal ideation and 6% prevalence for current suicidal behaviour. Of those with ideation (*n* = 47), nearly half (*n* = 21) thought of suicide often and very often. Of the 22 women who had made suicidal plans in the past month, 9 women had progressed to attempting suicide. Of those that attempted suicide in the past month, 5 had an intention to die while 4 hoped to be rescued/survive as defined by the MINI tool. It is important to note that all pregnant women that planned, prepared and/or attempted suicide also report suicidal ideation in the last month. Forty-one (11%) of all pregnant women in the sample had a lifetime suicide attempt.

Bivariate analyses indicated that of the pregnant women who reported any current SIB, 33% were diagnosed with MDE, 35% were diagnosed with some form of anxiety disorder and 54% had neither MDE nor anxiety disorder diagnosis as defined by the MINI Plus. Only four participants had AOD use diagnoses and SIB, while no participant was assessed to have ASPD. The distribution of risk factors according to suicide risk grouping is found in Table 2.

**Table 2:** Bivariate distributions

	Suicidal ideation (*n* = 47) (12%)	Suicidal behaviour (plan, intent and attempt) (*n* = 22) (6%)	Suicidal ideation and behaviour (*n* = 69) (18%)
MDE	15 (32)*	8 (37)	23 (33)**
Anxiety	16 (34)*	8 (37)*	24 (35)**
Lifetime suicide attempt	10 (21)*	8 (36)**	18 (26)**
Perceived support from significant other	Mean (23.6) SD (4.20)	Mean (21.4) SD (6.48)	Mean (22.9) SD (5.09)
Relationship type Married	17 (36)	6 (27)	**23 (33)**
Stable relationship	23 (49)	12 (55)	**35 (51)**
Casual relationship	4 (9)	2 (9)	**6 (9)**
Single/no relationship	3 (6)	2 (9)	5 (7)*
Intimate partner violence	9 (19)	9 (41)**	18 (26)**
Parity Nulliparous	**13 (28)**	**4 (18)**	17 (25)
Primiparous	**16 (34)**	**11 (50)**	27 (39)
Secundiparous	**8 (17)**	**5 (23)**	13 (19)
Multiparous	10(28)*	2 (9)*	12 (17)
Asset index Least poor	10 (21)	**10 (45)**	20 (29)
Poor	14 (30)	**1 (6)**	15 (21)
Very poor	11 (23)	**6 (27)**	17 (25)
Poorest	12 (26)	5 (23)*	17 (25)
Food insecurity	19 (40)	16 (73)**	35 (51)

Bold entries significant at *p* < 0.05

*Significant at ≤0.05; **Significant at ≤0.001

Result of the Cronbach’s α tests show that the MSPSS tool (Cronbach’s α 0.89), the CTS2 tool (Cronbach’s α0.85), and the HFSSM tool (Cronbach’s α 0.83) exhibit good internal consistency and reliability when used on the study sample. The multicollinearity test among independent variables also indicates that there was no multicollinearity within the regression model (max VIF = 1.41, condition number = 8.43).

Logistic regression analysis (Table 3) showed that sociodemographic and economic factors were strong predictors of current SIB. Multiple associations and co-existence of mental health disorders with SIB were observed among the sampled pregnant women. While women experiencing MDE were more likely to experience suicidal ideation only [aOR 1.86, 95% CI 1.69–3.65], women with any anxiety disorder diagnosis were more likely to experience suicidal behaviour [aOR 1.84, 95% CI 1.59–5.69]. MDE and any anxiety diagnosis predicted current suicidal ideation and current suicidal behaviour independently among pregnant women in the logistic regression analyses. However, in the adjusted model for SIB, these diagnoses became insignificant predictors. This we believe is a result of the existing moderate correlation between MDE and any anxiety disorder (correlation coefficient of 0.47). However, it is important to note that 67% of pregnant women that exhibited any current SIB had no MDE diagnosis, 65% had no anxiety diagnosis, while 54% has no MDE and anxiety disorders (Table 2).

**Table 3:** Multivariable regression output

	Suicidal ideation only (*n* = 47)		Suicidal behaviour (plan, intent, and attempt) (*n* = 22)		Suicidal ideation and behaviour (SIB) (*n* = 69)	
Risk factors	cOR (95% CI)	aOR (95% CI)	cOR (95% CI)	aOR (95% CI)	cOR (95% CI)	aOR (95% CI)
MDE	**1.86 (1.69–3.65)**	**1.42 (1.31–3.82)**	2.19 (0.88–5.44)	1.07 (0.31–3.62)	**2.14 (1.2–3.81)**	1.22 (0.59–2.51)
Anxiety	1.90 (0.98–3.68)	1.56 (0.70–3.43)	**2.02 (1.81–4.99)**	**1.84 (1.59–5.69)**	**2.10 (1.19–3.72)**	1.64 (0.82–3.27)
Lifetime suicide attempt (c9)	**2.59 (1.17–5.72)**	**2.33 (1.01–5.35)**	**5.55 (2.17–7.22)**	**5.92 (1.92–18.25)**	**4.35 (2.19–8.64)**	**4.01 (1.92–8.37)**
Perceived support from significant other	0.97 (0.90–1.04)	0.96 (0.89–1.04)	**0.88 (0.82–0.96)**	**0.91 (0.83–1.00)**	**0.92 (0.87–0.98)**	**0.93 (0.87–0.99)**
Relationship type Married	1	1	1	1	1	1
Stable relationship	1.03(0.52–2.01)	1.04 (0.50–2.13)	1.55 (0.56–4.24)	1.05 (0.33–3.33)	1.19 (0.66–2.12)	1.09 (0.58–3.48)
Casual relationship	2.52(0.73–8.73)	2.88 (0.74–11.19)	3.33 (0.61–18.10)	2.99 (0.43–21.24)	**3.20 (1.06–9.69)**	**3.69 (1.17–11.65)**
Single/no relationship	1.33 (0.35–5.05)	1.20 (0.29–4.92)	2.59 (0.48–13.82)	0.97 (0.10–8.78)	1.78 (0.58–5.38)	1.41 (0.42–4.69)
Intimate partner Violence	1.35 (0.61–2.97)	1.19 (0.50–2.79)	**4.30 (1.74–10.61)**	**2.41 (1.79–7.35)**	**2.35 (1.25–4.43)**	**2.14(1.10–4.19)**
Parity Nulliparous	1	1	1	1	1	1
Primiparous	1.19 (0.55–2.60)	1.20 (0.53–2.71)	2.77 (0.85–8.95)	2.32 (0.61–8.77)	1.65(0.84–3.12)	1.74 (0.87–3.48)
Secundiparous	0.89 (0.35–2.26)	0.87 (0.32–2.35)	1.89 (0.49–7.26)	1.58 (0.32–7.80)	1.14 (0.52–2.50)	1.35 (0.58–3.10)
Multiparous	**2.39 (1.96–5.94)**	**2.49 (1.90–6.82)**	1.37 (0.24–7.76)	0.70 (0.08–5.50)	2.24(0.97–5.18)	2.73(1.14–6.54)
Asset index Least poor	1	1	1	1	1	1
Poor	1.47 (0.61–3.49)	1.69 (0.67–4.23)	**1.90 (1.11–1.92)**	**1.07 (1.01–1.74)**	0.70 (0.33–1.47)	0.76 (0.34–1.71)
Very poor	1.08 (0.43–2.69)	1.24 (0.47–3.28)	0.56 (0.19–1.60)	0.74 (0.21–2.55)	0.79 (0.38–1.63)	0.94 (0.42–2.10)
Poorest	1.27 (0.52–3.11)	1.39 (0.54–3.57)	1.48 (1.16–1.59)	0.65 (0.18–2.29)	0.85 (0.41–1.75)	1.01 (0.45–2.21)
Food insecurity	0.92 (0.49–1.72)	0.59 (0.28–1.23)	**3.98 (1.52–10.41)**	**3.31 (1.04–9.21)**	**1.53 (1.49–2.60)**	1.03 (0.56–1.90)

Bold characters indicate significance at level *p* ≤ 0.05

Women who experienced a lifetime suicide attempt were twice as likely to experience suicidal ideation only [aOR 2.59, 95% CI 1.01–5.35], five times as likely to not only experience suicidal ideation but to plan, prepare and act on it [aOR5.92, 95% CI 1.92–18.25], and twice more likely to experience any current SIB [aOR 2.14, 955 CI 1.10–4.19]. As perception of social support from a significant other increases, pregnant women reported reduced odds of experiencing SIB compared to those that did not perceive social support [aOR 0.93, 95% CI 0.87–0.99].

Relationship characteristics and intimate partner violence were also explored as predictors of current SIB among the sampled pregnant women. Being in a casual, unmarried relationship placed women at greater risk of experiencing SIB relative to married women [aOR 3.69, 95% CI 1.17–11.65]. Women who experienced IPV were also twice as likely to experience suicidal behaviour [aOR 2.41, 95% CI 1.79–7.35], and two times more likely to experience SIB [aOR 2.14, 95% CI 1.10–4.19] compared to women that did not experience IPV. Participants who had more than two living children were twice as likely to report suicidal ideation only, compared to women with no living child [aOR 2.49, 95% CI 1.90–6.82].

Pregnant women who were food insecure were almost four times as likely to experience suicidal behaviours [aOR 3.98, 95% CI 1.52–10.41] than those who were food secure. Belonging to a lower socio-economic group as defined by an asset index placed participants at an increased risk for experiencing suicidal behaviours compared to the least poor [aOR 1.07, 95% 1.01–1.74].

## Discussion

With a 1-month prevalence of 18% for SIB, this study has confirmed high rates of suicidal ideation and behaviour among pregnant women living in adversity. This high prevalence is associated with co-existing mental health problems as well as socio-economic and demographic risk factors prevalent in the environment. A range of prevalence rates between 6 and 27.5%, using both diagnostic interviews and screening tools, have been reported for suicidal ideation among pregnant women living in adversity (Castro e Couto et al. 2016; Gausia et al. 2009; Huang et al. 2012; Lindahl et al. 2005; Rochat et al. 2013; Zhong et al. 2015).

Strong associations between MDE, anxiety disorder and suicide risk have been reported in both pregnant and non-pregnant women in LMICs (Cox et al. 2008; da Silva et al. 2012; Panagioti et al. 2012; Rochat et al. 2013). Although these studies utilise different assessment tools, with different cut-off points, findings underscore the elevated risk of SIB that pregnant women are exposed to when they experience any of the mentioned mental health problems and cohere with the results of this study. The studies cited mostly utilised screening tools which have typically been constructed to assess SIB to be a single phenomenon that is simply a symptom of mental illness. In our study, the MINI diagnostic tool afforded a more nuanced assessment of SIB. We were able to assess subcategories of SIB and were also able to assess frequency and intention. Further, these assessments were able to be made independently of diagnoses of mental health disorders.

There is an established body of literature which has shown a disproportionately high suicidal ideation among adults and pregnant women with comorbid depression and anxiety (Howard et al. 2011; Tabb et al. 2013; Siegel and Brandon 2014). However, our study found that more than half of the women that experienced SIB were not depressed nor diagnosed with any anxiety disorder. Our findings lend to the argument by Mann et al. (1999) and Oquendo et al. (2008) and suggest that suicidal ideation and behaviour should be screened for independently of mental disorders to avoid under-detecting individuals without these disorders, but who are at risk of suicide.

Association between lifetime suicide attempt and SIB has been documented in literature among general populations (Jones et al. 2003; Ballenger 2006) and adolescents (Cash and Bridge 2009; Guzmán et al. 2009), but with limited literature on pregnant women. However, these findings cohere with ours and indicate that these associations are exacerbated when there are comorbid mental health disorders (Jones et al. 2003) and other social risk factors including IPV (Cash and Bridge 2009).

A lack of social support has been shown to have a strong association with suicidal ideation, plan and attempt (Kleiner and Greston 1982) with increased likelihood when depression and low self-esteem co-exist (Liu and Mustanski 2012; Brausch and Decker 2014). A study among pregnant adolescents found that with increased perception of support from a partner and perceived approval from parents, teenage pregnant girls reported a lower frequency of suicidal thoughts (Brown et al. 2012). This was attributed to an improved outlook on life. The protective association between greater perceived social support and SIB in our study supports these findings.

It has been shown that partner status is strongly associated with suicidal ideation among pregnant women, particularly for those living without a partner (Huang et al. 2012; da Silva et al. 2012). Although these studies did not assess the association between having a casual relationship and SIB, our investigation revealed that women who had a casual relationship with their partner had an elevated risk for SIB. Casual relationships might also result in lower levels of perceived support. It has been shown that there are associations between having a partner, experiencing IPV, and experiencing depression and suicidal ideation (Gausia et al. 2009; McLaughlin et al. 2012). Further, severe violence is associated with a higher level of SIB, and IPV has also been found to be associated with incident suicidal ideation among women (Devries et al. 2013). These findings support our results which show a strong association between IPV and SIB and reveal that insecure relationships and IPV may create an environment which contributes to the development of SIB. Therefore, efforts should be made to address such factors through the integrated provision of IPV screening and management protocols (O’Campo et al. 2011) as well as therapeutic counselling modalities such as Interpersonal Therapy (Bolton et al. 2003).

1Poverty, in the form of food insecurity, was also found to predict SIB in this study. A South African study among postnatal women attending a community-based child and nutrition programme found that over half of their study population (59.8%) were food insecure and that food insecurity created an elevated probability of suicidal ideation, depression and alcohol abuse (Dewing et al. 2013). Similarly, lower socio-economic status predicted SIB among these women. Similar associations between food insecurity and SIB among patients attending HIV clinics in Uganda (Kinyanda et al. 2012) and self-reported suicide attempts among women in India (Maselko and Patel 2008) have been found. Our findings thus further endorse the argument in favour of protecting impoverished women and their infants, through social infrastructures like social support. This highlights the importance of eco-systemic suicide prevention programmes which move beyond simply identify and providing care to individuals with diagnosed mental disorders.

Our study experienced several limitations in design. Firstly, the cross-sectional nature of the data limits an investigation into progression of SIB and other mental health disorders prior to conception. We were also not able to track the long-term suicide-related outcomes of the sample. The study sample size was relatively small and hence generalisability of findings to other populations should occur with caution. All data collected was self-reported and we did not verify information such as asset ownership and prior self-reported mental health diagnosis. Thus, there is room for recall bias and misreporting of information. Further, although there is a high uptake (97%) of antenatal services in South Africa (Shisana et al. 2010), data was collected at clinic level. Therefore, non-users of antenatal services who might be the most vulnerable to suicidal ideation and behaviour may have not been included in the sample. HIV data was collected retrospectively, and hence, we could not investigate the effect of HIV on suicidal ideation. Finally, the MINI diagnostic tool assesses for only one type of personality disorder (antisocial personality disorder) which limits the assessment of potential associated risk of some personality disorders, especially borderline personality disorder, with SIB. The number of pregnant women with SIB and no MDE or anxiety may have these personality disorders although none of our study participants were assessed to have antisocial personality disorder. However, in light of these limitations, this study exhibits the strength as one of the few studies that have investigated the prevalence, diagnostic comorbidities and associated risk factors of suicidal ideation and behaviours among low-income pregnant women in Sub-Sahara Africa.

In summary, our study has shown a high prevalence of SIB among pregnant women living in adversity. The presence of comorbid MDE and anxiety, together with other sociodemographic, economic and psychosocial risk factors, illustrates the elevated vulnerability of these women. It is noteworthy that although global evidence reports depression to be strongly associated with suicidal ideation, the majority of pregnant women with SIB were not depressed in our study. This has implications for screening for mental health disorders in pregnant women. Commonly used depression screening tools have suicide-related questions imbedded within their scoring systems. Hence, there is a risk of under-detection of SIB in women who do not have common perinatal mental disorders, as further assessment and intervention usually occurs for those who meet the cut-off for these disorders on the screening scales. Thus, we recommend that further investigation establishes how best to identify women with SIB and that SIB should be assessed independently of common mental disorders.

Our study supports the findings that SIB is fairly common among pregnant women. We propose that public health and social interventions should address the underlying psychosocial risk factors for high-risk SIB among economically and socially vulnerable women in the perinatal period. Further, we suggest longitudinal studies to investigate the progress of suicidal ideation through thought, planning and attempt, among these vulnerable groups. A better understanding of the relationship between SIB and associated risk factors would assist in the development of detection instruments and interventions aimed at addressing holistically, the needs of vulnerable pregnant women. Also, towards these goals, we recommend that future studies investigate the circumstances whereby SIB may exist independently of MDE and anxiety disorders.
